# Whole-Genome Sequence of Acetobacter orientalis Strain FAN1, Isolated from Caucasian Yogurt

**DOI:** 10.1128/genomeA.00201-18

**Published:** 2018-03-29

**Authors:** Nobutaka Nakashima, Tomohiro Tamura

**Affiliations:** a Bioproduction Research Institute, National Institute of Advanced Industrial Science and Technology (AIST), Toyohira-ku, Sapporo, Japan; b Laboratory of Molecular Environmental Microbiology, Graduate School of Agriculture, Hokkaido University, Kita-ku, Sapporo, Japan; c Computational Bio Big Data Open Innovation Laboratory (CBBD-OIL), AIST, Toyohira-ku, Sapporo, Japan

## Abstract

In traditional Caucasian yogurt, Acetobacter orientalis bacteria play important roles in the fermentation of milk in concert with Lactococcus bacteria. In this study, an A. orientalis strain, FAN1, was newly isolated from commercially available Caucasian yogurt, and its whole-genome sequence was determined, identifying two circular DNAs.

## GENOME ANNOUNCEMENT

Acetobacter orientalis belongs to the class *Alphaproteobacteria* and is an aerobic rod-shaped bacterium (1). A. orientalis cells have been isolated from canna flower, starfruit, coconut, the curds of tofu, tempeh, and traditional Caucasian yogurt (called Caspian Sea yogurt) (2, 3). In Caucasian yogurt, milk ingredients are mainly fermented by Lactococcus lactis subsp. cremoris cells; however, coexisting A. orientalis cells are also important for stable fermentation (1, 3). Because A. orientalis cells in Caucasian yogurt have the ability to produce lactobionic acid, which is useful to the food and pharmaceutical industries, these cells may represent a suitable host for producing food-grade lactobionic acid (1).

Here, we present the whole-genome sequence of A. orientalis strain FAN1, which was newly isolated from commercially available Caucasian yogurt (Fujicco Co., Ltd., Kobe, Japan). Yogurt was thinly streaked on BBL standard methods agar (Becton, Dickinson, MD, USA), forming some isolated colonies. A part of the 16S rRNA gene (760 bp) was amplified by PCR from one colony, and the sequence of the DNA fragment was determined using Sanger sequencing; from this analysis, the colony was identified as A. orientalis. The identified colony was further cultured in liquid de Man-Rogosa-Sharpe (MRS) broth (Difco Laboratories, Detroit, MI, USA). Whole-genome DNA was isolated using the neutral phenol-chloroform-isoamyl alcohol method (4) and was subjected to sequencing with PacBio RS II and Illumina HiSeq 2500 systems (Hokkaido System Science Co., Ltd., Sapporo, Japan). The complete genome sequence was assembled using the SMRT Pipeline (HGAP.2) program (5), followed by gene annotation using the Glimmer, RNAmmer, tRNAscan-SE, BLAST, and Blast2GO programs (6–10).

The FAN1 strain was found to have two circular DNAs, with lengths of 3,041,114 and 173,853 bp. Based on its length, the larger DNA was assumed to be the chromosomal DNA, and the smaller DNA was expected to be a plasmid. The gene annotation programs detected 3,563 coding sequences; among these sequences, 3,345 coding sequences were on the chromosomal DNA. In addition, 15 rRNAs (five 23S, 16S, and 5S rRNAs each) and 55 tRNAs were detected on the chromosomal DNA. The G+C contents of the DNAs were 52.34% and 55.96%, respectively.

### Accession number(s).

The entire sequence has been deposited in DDBJ/ENA/GenBank under accession numbers AP018515 and AP018516. The version described in this paper is the first version.
